# Changes in the population and community structure of corals during recent disturbances (February 2016-October 2017) on Maldivian coral reefs

**DOI:** 10.1038/s41598-019-44809-9

**Published:** 2019-06-10

**Authors:** C. Pisapia, D. Burn, M. S. Pratchett

**Affiliations:** 1 Department of Biology, California State University of Northridge, 18111 Nordhoff Street, Northridge, 91330-8303 California, USA; 2 ARC Centre of Excellence for Coral Reef Studies, James Cook University, QLD 4811 Townsville, Australia

**Keywords:** Climate-change ecology, Population dynamics

## Abstract

Climate change is the greatest threat to coral reef ecosystems. In particular, increasing ocean temperatures are causing severe and widespread coral bleaching, contributing to extensive coral loss and degradation of coral reef habitats globally. Effects of coral bleaching are not however, equally apportioned among different corals, leading to shifts in population and community structure. This study explored variation in bleaching susceptibility and mortality associated with the 2016 severe mass bleaching in the Central Maldives Archipelago. Five dominant coral taxa (tabular *Acropora*, *Acropora humilis*, *Acropora muricata*, *Pocillopora* and massive *Porites*) were surveyed in February 2016 and October 2017 to test for changes in abundance and size structure. Substantial taxonomic differences in rates of mortality were observed; the most severely affected taxa, *Acropora*, were virtually extirpated during the course of this study, whereas some other taxa (most notably, massive *Porites*) were relatively unaffected. However, even the least affected corals exhibited marked changes in population structure. In February 2016 (prior to recent mass-bleaching), size-frequency distributions of all coral taxa were dominated by larger size classes with over-centralized, peaked distributions (negatively skewed with positive kurtosis) reflecting a mature population structure. In October 2017, after the bleaching, coral populations were dominated by smaller and medium size classes, reflecting high levels of mortality and injury among larger coral colonies. Pronounced changes in coral populations and communities in the Maldives, caused by coral bleaching and other disturbances (outbreaks of crown-of-thorns starfish and sedimentation), will constrain recovery capacity, further compounding upon recent coral loss.

## Introduction

Climate change is among the foremost threats to natural ecosystems, contributing to species transformations and global degradation across many different aquatic and terrestrial habitats^1–3^. Coral reefs are one of the most vulnerable ecosystems to climate change^4,5^, wherein increasing ocean temperatures, increased intensity of severe tropical storms (e.g., cyclones), and emerging effects of ocean acidification, are contributing to new ecological configurations (or phase shifts) and sustained declines in complexity and diversity of reef habitats. Relatively recent and rapidly accelerating effects of climate change^6^ are occurring against a backdrop of long-term, system-wide changes in the structure and function of reef ecosystems, caused by sustained exploitation of reef species, as well as sedimentation, eutrophication and pollution from coastal development^7,8^. The history of disturbances and pressures structuring coral reefs may make reef assemblages even more vulnerable to climate change^9^ due to compromised health and physiological challenges^10^, increased prevalence of highly susceptible species^11^, and declines in abundance of functionally-important species that contribute to ecosystem resilience^12^. Moreover, climate change will compound upon pre-existing disturbances and pressures to undermine key ecosystem processes (e.g., calcification^13^), as well as the goods and services derived from coral reef ecosystems (e.g., fisheries production^14^).

The foremost effect of anthropogenic climate change on coral reefs is large-scale multi-specific coral bleaching (*mass bleaching*^15^), which is caused by elevated ocean temperatures^16,17^. Coral bleaching results from a breakdown in the relationship between the coral host and their photosynthetic endosymbionts (Symbiodiniaceae), caused by a wide range of environmental stresses, including elevated temperatures, low salinity or extreme exposure to ultraviolet light^16,18^. However, increasing incidence and severity of mass coral bleaching is incontrovertibly linked to climate induced ocean warming^6^. Bleached corals are compromised both nutritionally and physiologically, and often die when subject to severe or prolonged bleaching^19,20^. Increases in the global incidence of mass bleaching, along with perennial threats to corals, are already leading to alterations in the structure and function of reef ecosystems^21^.

Bleaching susceptibility greatly varies among different coral taxa^22–25^, whereby the portion of colonies that bleach and die is invariably higher for some coral taxa (e.g., *Acropora* and *Stylophora*), whereas other taxa (e.g., *Galaxea* and *Cyphastrea*) rarely exhibit coral bleaching, except during the most extreme heatwaves^22–25^. These taxonomic differences will contribute to changes in the community structure of coral assemblages^23,26^, though it is both differential susceptibility and recovery that will determine the future structure (and vulnerability) of coral assemblages^27^. Rapid recovery of coral assemblages is largely achieved by proliferation of fast growing coral species (e.g., *Acropora*^28^), which may make coral assemblages even more vulnerable to subsequent disturbances.

Inter-specific variation in bleaching susceptibility has been attributed to differences in Symbiodiniaceae density and/or clades^29^, heterotrophic plasticity^30^, depletion of energy reserves^20^, and physiological and/or morphological differences among coral species^22,23^. There is also evidence for size-based variation in bleaching susceptibility where juveniles and smaller colonies can withstand higher temperatures than larger colonies^23,25,31–33^, potentially due to differences in flow regime and mass transfer of detrimental photosynthetic byproducts^32^. Moreover, coral bleaching may greatly alter the size-structure of coral populations, both due to differential susceptibility of large versus small colonies and incomplete or partial mortality that effectively reduces the size of colonies. Massive *Porites*, for example, may survive severe bleaching events^34^, but experience high rates of partial mortality that cause reductions in the size of colonies, thereby affecting growth, regeneration and reproduction^35,36^. Larger colonies also contribute disproportionately to recovery due to inherent size-based differences in reproductive potential, and the capacity to withstand further disturbances^34^. With escalating effects of global climate change, it is expected that coral populations will be increasingly dominated by small colonies and have faster turnover^23,32,37^. Size-specific bleaching susceptibility may therefore, have severe consequences for population replenishment. While survival of juvenile corals is beneficial for recovery^38^, loss of larger colonies may constrain reproduction following bleaching^36,39^.

In 2015–2017, extreme temperature anomalies triggered unprecedented mass coral bleaching, which not only affected virtually all major coral reef regions throughout the world, but also caused successive years of mass bleaching in many regions^13,17,40,41^. This latest global mass bleaching event exceeded the scale and severity of the 1998 mass bleaching, which killed >90% of shallow–water corals on many reefs, especially in the western Indian Ocean^42,43^. In the Maldives, located in the north western Indian Ocean, coral cover declined to 1–8% in the aftermath of the 1998 coral bleaching event, down from 40–60% coral cover prior to this event^42,44^. Despite subsequent disturbances, including moderate coral bleaching in 2010, coral assemblages largely recovered in the aftermath of the 1998 coral bleaching, increasing to >40% in 2015^44^.

**Figure 1:**
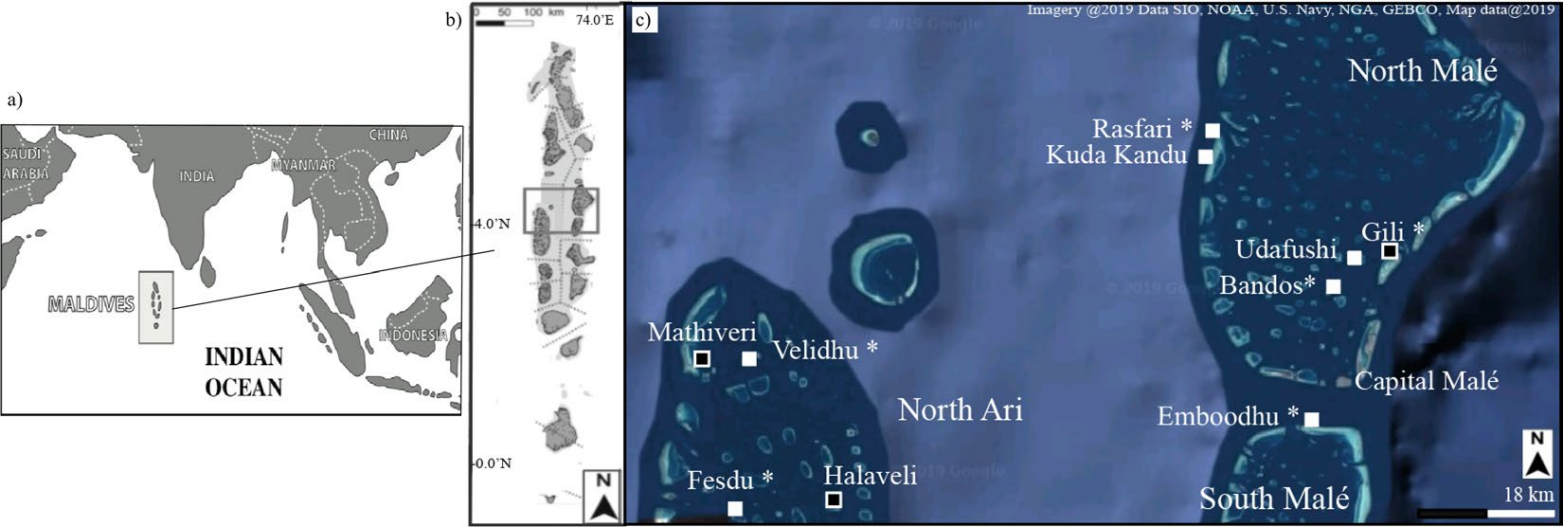
Map of the study sites in the Central Maldives Archipelago, Indian Ocean inclusive of (**a**) the global map of the Indian Ocean, (**b**) an enlargement of the Maldives and (**c**) the area of study. White squares are the sites surveyed in both 2016 and 2017, while black squares refer to sites surveyed only in 2017. Site and atoll names are reported. The symbol * refers to sites also affected by outbreaks of *A*. *planci* and/or sedimentation. The oceanic sites (KudaKandu, Rasfari, and Emboodhu) were all on the fore-reefs. This figure was generated using Google Earth Digital Globe (https://earth.google.com), and Adobe Illustrator and was modified after^73^.

**Figure 2:**
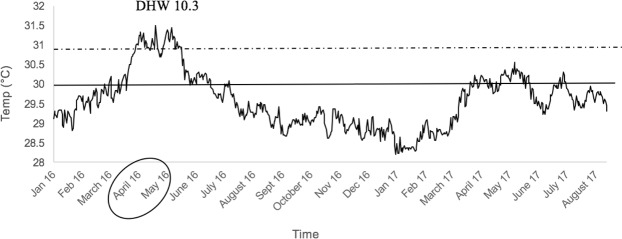
Daily temperatures (°C) from January 2016 to August 2017 in the Central Maldives Archipelago. The continuous line indicates maximum monthly mean SST, while the dotted line indicates local bleaching threshold SST. Data were downloaded from: https://coralreefwatch.noaa.gov/vs/gauges/maldives.php. The NOAA estimate of degree heating weeks (DHW) peaked is 10.443 at June 26, 2016.

This study explores the effects of the latest (2015–2017) mass bleaching event on coral assemblages in the Central Maldives Archipelago (Fig. 1). Coral bleaching in the Maldives was most pronounced in May-June 2016 (NOAA, 2016;^13^, driven by strong ENSO-induced warming and elevated temperatures that persisted from March 2016 through until mid-May 2016 (NOAA, 2016; Fig. 2). Importantly, coral assemblages in the Central Maldives were not only affected by the mass bleaching but also by other localized disturbances, including outbreaks of crown-of-thorns starfish (*Acanthaster planci*) and sedimentation^44^. To assess the effects of the mass bleaching and other localized disturbances, the abundance and size-structure of dominant coral taxa were quantified in February 2016 (several months before the bleaching) and then again in October 2017 (>12-months following the severe bleaching event), within 2 depth strata (5 m and 10 m) at each of 10 locations throughout the Central Maldives Archipelago. Temporal comparisons of the abundance and size structure provided important information on size-specific susceptibility and mortality of coral populations, which is critically important in understanding future effects of climate change on coral assemblages^6^. We expected that: (i) recent disturbances (e.g., bleaching, *A*. *planci* outbreaks and sedimentation) would have disproportionate effects on larger coral colonies^31–33,35,36^, resulting in marked shifts in coral size structure with increasing dominance of smaller size classes, and (ii) there would be shifts in taxonomic composition, reflective of differential taxonomic susceptibility to coral bleaching and other recent disturbances^22–25^. More specifically, we expected that *Acropora*, which was the predominant habitat forming taxa at many of the study sites in 2016^44^, would be disproportionately affected by recent disturbances in the Maldives, leading to increased dominance of massive and robust taxa. If so, this is likely to have important consequences for the function and resilience of coral assemblages^28^. The size, abundance and identity of juvenile corals was also quantified in the aftermath of the recent mass bleaching and other disturbances, to better understand the potential for population replenishment and recovery. We expected that contemporary densities of juvenile corals, reflective of rates of recent settlement and post-settlement survivorship, would be suppressed in the aftermath of the recent mass bleaching^39^. If so, this would greatly reduce rates of recovery for coral assemblages in the Maldives.

## Results

### Changes in coral cover

**Figure 3:**
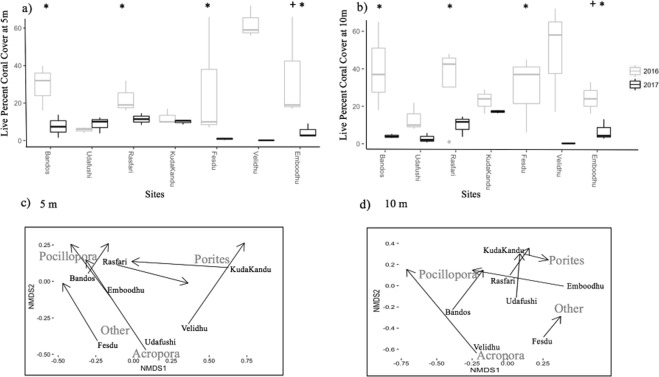
Variation in mean (±SE) live coral cover at (**a**) 5 and (**b**) 10 meters in all study sites before and after the bleaching; the * refers to site affected by outbreaks of *A*. *planci*, while the + refers to sites affected by sedimentation; (**c**) nMDS graph used as visualization tool to show shifts in coral assemblages at each site between February 2016 and October 2017 at 5 m (stress = 0.081) and (**d**)10 m (stress = 0.096). The black arrows connect the 2016–2017 pairs of data points for each site to show changes in adult composition after eighteen months. nMDS stress is specified for each graph (**c**,**d**).

Between February 2016 and October 2017, coral cover declined substantially across the seven study sites in three atolls in the Central Maldives Archipelago (Fig. 1). Mean coral cover in shallow (5 m) habitats declined from 29.4% (±6.94 SE) down to 5.6% (±1.22 SE). In deeper (10 m) habitats, coral cover went from 28.3% (±5.83 SE) in February 2016 down to 5.9% (±1.91 SE) in October 2017 (Fig. 3). Coral loss coincided with, and was at least partially attributable to, extreme ocean temperatures (up to 31.5 °C recorded in April and May 2016: Fig. 2) and severe coral bleaching recorded from March 2016. However, there were also other localised disturbances, including sedimentation from land reclamation and ongoing outbreaks of *Acanthaster planci* (Fig. 3), which contributed to coral loss at some locations^44^. Most notably, there was extensive dredging and sedimentation associated with land reclamation along the northern edge of South Male Atoll, which was very apparent at Emboodhu. There were also outbreaks of *A*. *planci* recorded in parts of the Maldives (mostly at North Male Atoll, but also Ari Atoll), that caused extensive coral loss even before the recent mass-bleaching^44^. Outbreaks were mostly waning through the course of this study, though moderate densities of *A*. *planci* and evidence of recent coral predation were recorded mainly at Rasfari and Fesdu, but also at Bandos and Emboodhu.

In February 2016 (before the recent mass-bleaching), average coral cover varied among the seven study sites (Fesdu and Velidhu at North Ari Atoll; Bandos, Kuda Kandu, Rasfari and Udhafushi at North Male Atoll; Emboodhu at South Male Atoll), ranging from 6% (±0.88 SE) at Udhafushi, up to 62.6% (±4.91 SE) at Velidhu, in the shallow (5 m) reef habitat (Fig. 3). Regardless of initial coral cover, coral cover recorded in October 2017 was <11% at virtually all study sites, except for Kuda Kandu, where coral cover was >17% at 10 m (Fig. 3). There was also limited change in coral cover between surveys (February 2016 to October 2017) at Kuda Kandu, either for shallow (5 m) or deeper (10 m) habitats (Fig. 3). Elsewhere, coral loss ranged from 48.8% at Rasfari to 99.95% at Velidhu (Fig. 3). The worst affected site was Velidhu, located in North Ari Atoll, where coral cover declined from 62.6% to 0.03% and 49.3% to 0.07%, at 5 m and 10 m, respectively. Importantly, the coral assemblages at Velidhu in February 2016 were dominated by *Acropora* (40.6% at 5 m, while it was lower than 15% at other sites) (Fig. 3), in particular, *A*. *muricata*, which probably accounted both for the higher coral cover, but also increased vulnerability to subsequent disturbances. Despite variation in reef habitats across the Central Maldives, based on island position and exposure, there was no apparent difference in the extent of coral loss recorded at sites on the outer side of the atolls compared to inner sites (Table 1). It is however possible, that increased sampling may have revealed significant differences between oceanic and lagoon sites. Different Generalized Least Squares (GLS) models were compared to investigate the significance of different predictors such as island exposure, site, depth, and year, including fixed effects and their interactions (Table 1). While adding depth and island exposure as variables to the model did result in better explanatory power (*χ*^2^ = 36.9, *df* = 5, *p* < 0.01), it also resulted in a higher AIC value (Table 1). Adding only site and year as variables significantly improved the model compared to the null (*χ*^2^ = 12.18, *df* = 9, *p* < 0.01) and resulted in the model with the lowest AIC (Table 1). The best model indicated that coral loss changed dramatically from 2016 to 2017 (df = 80, t-value = −7.03, Pr < 0.01) and that change was consistent across all sites, except Udafushi (df = 80, t-value = −2.07, Pr = 0.04) (Table S4).

**Table 1:** The performance of GLS and GLMM to explain changes in coral cover and juvenile abundance, showing Akaike Information Criterion (AIC) and Log Likelihood (LogLik) for all the models. The models used in the analyses are highlighted in bold.

*Generalized Least Squares models*	AIC	LogLik
model.null <- gls (Coral Cover ~1)	704.3275	−350.1638
model.1 <- gls (Coral Cover ~ Island Exposure + Depth)	707.7458	−349.8729
model.2 <- gls (Coral Cover ~ Island Exposure + Depth + Year)	672.8551	−331.4276
model.3 <- gls (Coral Cover ~ Island Exposure + Depth * Year)	674.6118	−331.3059
**model**.**4 <- gls** (**Coral Cover ~ Year + Site**)	**668**.**4297**	**−325**.**2148**
model.5 <- gls (Coral Cover ~ Site)	708.2892	−346.1446
model.6 <- gls (Coral Cover ~ Year)	669.4592	−331.7296
model.7 <- gls (Coral Cover ~ Site + Depth + Year)	669.8657	−324.9328
model.8 <- gls (Coral Cover ~ Island Exposure)	706.3261	−350.163
model.9 <- gls (Coral Cover ~ Depth)	705.7513	−349.8756
***Generalized Linear Mixed Model with negative binomial***	**AIC**	**LogLik**
model.null <- nb(Juvenile Abundance ~ (1|Site))	2298.1	−1146.1
**model**.**1 <- nb**(**Juvenile Abundance ~Depth + **(**1|Site**))	**2293**.**1**	**−1142**.**5**
model.2 <- nb(Juvenile Abundance ~Depth + Island Exposure + (1|Site))	2295.0	−1142.5
model.3 <- nb(Juvenile Abundance ~Island Exposure + (1|Site))	2300.0	−1146
model.4 <- nb(Juvenile Abundance ~Depth* Island Exposure + (1|Site))	2294.1	−1142.1

**Figure 4:**
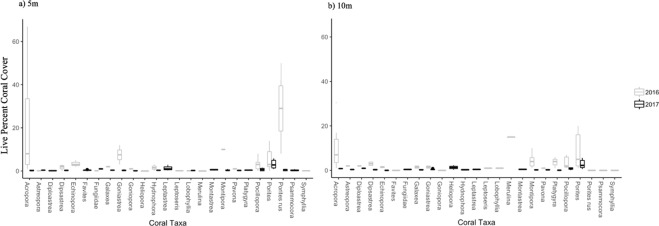
Variation in mean (±SE) live coral cover of different coral taxa at (**a**) 5 m and (**b**) 10 m sites before and after the bleaching.

Recent disturbances caused marked transformations in coral assemblages at the study sites (Figs 3, 4), caused mainly by disproportionate declines in the formerly dominant coral taxa, *Acropora* (Figs 3c,d, 4). *Montipora*, *Echinopora* and *Goniastrea* also showed severe declines in abundance (Fig. 4). By contrast, there were only moderate declines in abundance of *Pocillopora* and *Porites* and these became increasingly dominant due to disproportionate losses of aforementioned taxa (Figs 3, 4). Species composition of less abundant taxa such as *Dipsastrea*, *Goniopora* and *Hydnophora* was also altered from 2016 to 2017 (Fig. 4). The degree of change differed among sites with some sites like Bandos and Rasfari showing smaller changes in coral composition compared to others such as Velidhu (Fig. 3c,d). Overall, shifts in live cover of different coral taxa were consistent between depths (Figs 3c,d, 4).

### Changes in size-frequency distributions

Comparisons of population structure for each of the five focal taxa (tabular *Acropora*, *A*. *muricata*, *A*. *humilis*, *Pocillopora* and massive *Porites*) were constrained by the very limited abundance of some corals in October 2017 (Table 2). Most notably, densities of tabular *Acropora*, *A*. *muricata* and *A*. *humilis* were <10% of that recorded in February 2016, reflecting widespread declines in abundance of these corals (Table 2). Densities of *Pocillopora* and massive *Porites* were also lower in October 2017, compared to February 2017, though the absolute number of colonies recorded in 2017 was far greater than recorded for the *Acropora* corals (Table 2).

**Table 2:** Statistical summary of log-transformed size-frequency distributions for each coral taxon at 5 m and 10 m before the mass bleaching in 2016 and following the event in 2017.

Year	Species	Depth (m)	n	Mean colony size (cm^2^)	CV	Kurtosis	Skewness
2016	*Acropora muricata*	5	104	3.6	21.6	3.4	−0.4
2016	*Acropora humilis*	5	95	2.8	20.8	2.3	0.1
2016	Tabular *Acropora*	5	58	3.7	27.6	1.8	−0.1
2016	*Pocillopora* spp	5	283	2.7	26.6	2.4	−0.3
2016	*Porites*	5	421	2.9	18.8	2.9	−0.06
2016	*Acropora muricata*	10	95	3.5	23.9	2.8	−0.1
2016	*Acropora humilis*	10	67	2.7	17.6	2.8	−0.2
2016	Tabular *Acropora*	10	24	3.9	18	2.1	−0.6
2016	*Pocillopora* spp	10	238	2.8	25.03	2.3	−0.6
2016	*Porites*	10	546	2.9	19.5	3.9	−0.6
2017	*Acropora muricata*	5	0				
2017	*Acropora humilis*	5	10	184.9	103.5	2.08	0.9
2017	Tabular *Acropora*	5	21	1.7	21.4	3.6	1.2
2017	*Pocillopora* spp	5	218	1.9	30.3	2.05	0.37
2017	*Porites*	5	452	2.5	17.8	3.1	−0.08
2017	*Acropora muricata*	10	4				
2017	*Acropora humilis*	10	6	2.1			
2017	Tabular *Acropora*	10	3				
2017	*Pocillopora* spp	10	108	2.1	30.2	1.7	−0.1
2017	*Porites*	10	364	2.5	18.1	3.1	−0.1

The sample size (n), log-transformed mean colony size, coefficient of variation (CV), kurtosis (g_2_) and skewness (g_1_) are specified.

**Figure 5:**
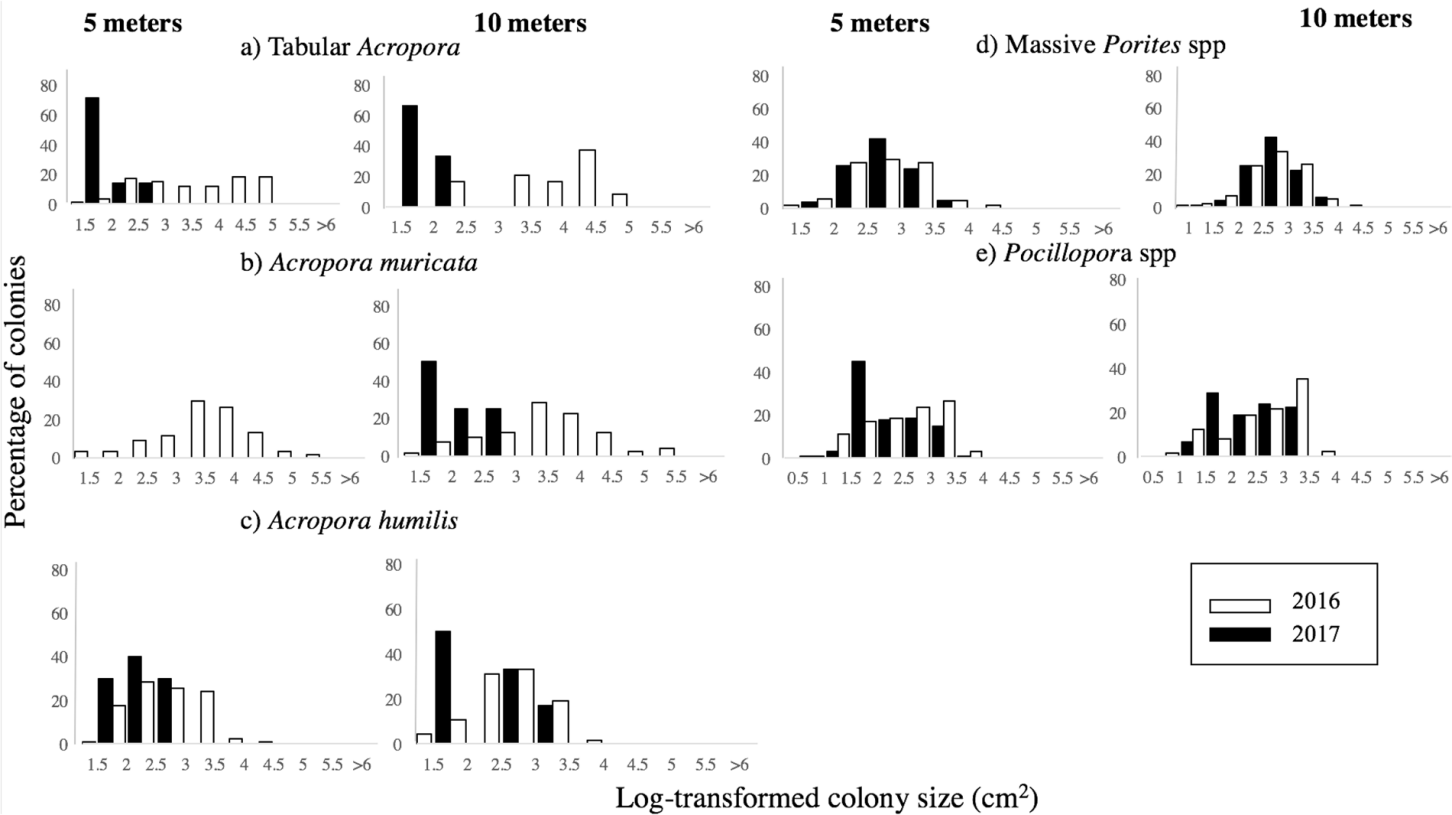
Log-transformed size-frequency distributions of *Acropora muricata*, tabular *Acropora*, *Acropora humilis*, *Pocillopora* spp and *Porites* spp at 5 m and 10 m before and after the mass bleaching event.

Aside from marked declines in the abundance of all corals, there were apparent changes in the structure of coral populations between February 2016 and October 2017 (Fig. 5). In February 2016 (before the bleaching), there was a preponderance of colonies in the largest size-classes for all taxa at both 5 and 10 m, where transformed size-frequency distributions were negatively skewed (Fig. 5, Table 2). Size-frequency distributions were also leptokurtic, peaked and highly centralized around the mean, indicative of a mature population structure (Fig. 5, Table 2). Conversely in 2017, untransformed data shows a large prevalence of smaller colonies in all coral taxa at both depths resulting in positively skewed size-frequency distributions (Fig. S1). Even log-transformed size-frequency distributions were positively skewed for most coral taxa, with a predominance of smaller size classes (Fig. 5, Table 2). Size-frequency distributions in all taxa showed positive kurtosis similar to pre-bleaching states (i.e., leptokurtic), they were peaked and highly centralized around the mean. In 2016, a high percentage (62%) of *A*. *muricata* colonies were in the largest class size (>10,000 cm^2^), whereas all colonies recorded in 2017 were in the smallest size classes (Fig. 5). Given that there was an increase in the absolute number of colonies in these small size classes, it is likely that larger colonies were effectively reduced in size through partial mortality or fission (Fig. 5). All coral taxa in 2017 showed similar size-frequency distributions at both depths (2- sample Kolmogorov–Smirnov (KS) tabular *Acropora* D = 0.38 p = 0.8, *Acropora humilis* D = 0.23 p = 0.98, *Porites* spp D = 0.07 p = 0.27), except *Pocillopora* spp (2- sample Kolmogorov–Smirnov (KS) D = 0.15, p = 0.05), which showed higher abundance of smaller colonies at 5 m (Fig. 5).

Changes in size structure were further reflected in changes in mean size (measured based on live surface area (SA)) of corals between 2016 and 2017 for all focal taxa [One-way ANOVA: *Porites* (F_1/1772_ = 177.8, p < 0.001), tabular *Acropora* (F_1/99_ = 11.7, p < 0.001), *Acropora humilis* (F_1/168_ = 3.09, p < 0.001) and *Pocillopora* (F_1/834_ = 105.4, p < 0.001)]. Importantly, surface area in each coral taxon did not vary between depths (except tabular *Acropora*), but varied among sites (except *A*. *humilis*) (Table 3).

**Table 3:** Statistical summary of two-way Anova for 4 coral taxa tested in 2017 using mean surface area per transect, site, and depth as variables.

	df	SS	F	p
***Acropora humilis***				
Site	3	140387	3.2	0.14
Depth	1	438	0.03	0.9
Error	4	58438		
***Acropora plate***				
Site	2	45845	166.7	***
Depth	1	6289	45.73	0.02
Error	2	275		
***Pocillopora*** **spp**				
Site	9	3592636	8.18	***
Depth	1	186412	3.82	0.06
Error	32	1560830		
***Porites***				
Site	8	1727778	5.98	***
Depth	1	113044	3.12	0.08
Error	36	1300600		

There were not enough colonies of *A*. *muricata* to run any analyses. The three *** refer to p < 0.001.

### Juvenile density after recent disturbances

**Figure 6:**
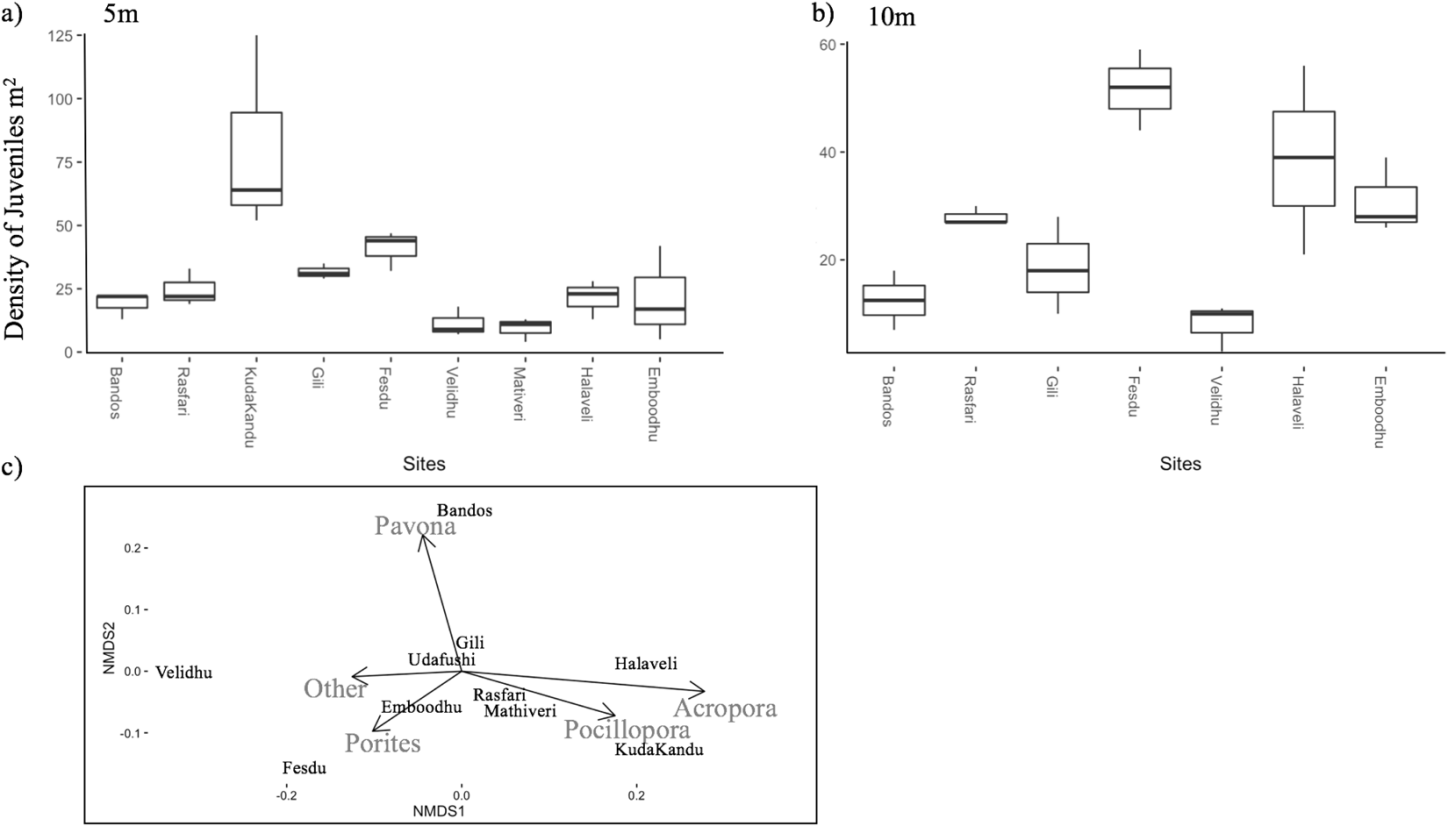
Relative abundance of juvenile *Acropora*, *Pocillopora*, *Porites*, *Pavona* and other corals among the study sites at (**a**) 5 m and (**b**) 10 m in 2017. (**c**) nMDS graph used as visualization tool to show composition of juvenile corals in October 2017 following major disturbances (stress = 0.039). nMDS and stress are merely used as visualization tools.

A total of 1,643 juvenile corals were counted across all sites in October 2017 (n = 57 transects), corresponding with a mean density of 2.88 (±0.97 SE) juvenile corals per m^2^. *Pavona* and *Porites* accounted for the majority of juveniles, accounting for 21.05% (356/1643) and 19.05% (313/1643) of juveniles, respectively, pooled across depths (Figs 6, S2). Juvenile coral assemblages were dominated by *Pavona* (Figs 4, 6, S2) though this taxon accounted for a small proportion (<5%) of adult coral cover. Juvenile *Porites* were relatively abundant at all sites, but particularly so at Fesdu, where >50% of juvenile corals were *Porites* (Figs 6, S2). *Acropora* and *Pocillopora* were relatively underrepresented in the juvenile coral assemblages (Figs 6, S2), except at Kuda Kandu, where there were high densities of juveniles of both *Acropora* and *Pocillopora* (3.7 ± 5.17 SE and 3.4 ± 3.63 SE per m^2^, respectively) in the shallow reef habitat (Figs 6, S2). Elsewhere, densities of *Acropora* and *Pocillopora* were mostly <2 juveniles per m^2^ (Figs 6, S2). Analyses based on a Generalized Linear Mixed Model (GLMM) suggested that overall juvenile abundance did not vary among sites (random effect Variance = 0.09, Standard deviation = 0.31), but varied with depth (df = 503, z-value = −2.67, Pr = 0.007), whereby, juveniles were more abundant at 5 m (Figs 6, S2). Importantly, in the GLMM, adding island exposure and depth as predictors resulted in good explanatory power (*χ*^2^ = 7.02, *df* = 1, *p* = 0.008), and similarly adding only depth also significantly improved the model compared to the null (*χ*^2^ = 7.08, *df* = 1, *p* = 0.007), however, the model with only depth as predictor resulted in the lowest AIC (Table 1).

## Discussion

Coral reefs are being increasingly affected and altered by global climate change^6,21,39^. Most critically, ocean warming and associated increases in the incidence and severity of mass coral bleaching are undermining the functional importance of corals in maintaining reef structures^13^, as well as altering the composition of coral assemblages^21^. Sustained periods of elevated ocean temperatures in 2016 and 2017 have caused extensive bleaching in most coral reef regions^6^, including the western Indian Ocean. Peak temperatures recorded in the Maldives in 2016 were well above established bleaching thresholds (Fig. 2^13^) and the elevated temperatures occurred over an extended period, resulting in cumulative heat stress that greatly exceeded 8 degree heating weeks (DHW), which is the level at which widespread coral bleaching and mortality is expected^45^. Not surprisingly, severe coral bleaching was recorded throughout much of the Maldives, from March 2016^46,47^. Our surveys reveal that there was substantial coral loss between February 2016 and October 2017 at many sites, reflecting the severity of the thermal stress and coral bleaching. However, coral mortality caused by coral bleaching was compounded and/or exacerbated by other localized disturbances, such as sedimentation and ongoing (though waning) outbreaks of crown-of-thorns starfish^48^. Coral loss was generally equivalent between the reef crest (5 m depth) and reef slope (10 m depth), though there is the potential that much deeper reef habitats might have been spared from the worst effects of elevated ocean temperatures and associated bleaching^47^.

Mass bleaching conditions are generated by large-scale increases in ocean temperatures, but bleaching responses and subsequent coral mortality can be extremely patchy^49^. Small-scale differences in bleaching incidence and severity may be due to differences in light and flow^32^, but also varies according to the structure of local coral assemblages^17^. Spatial variation (among sites) in coral loss in the Maldives partly reflects differences in the predominance of corals that are particularly susceptible to coral bleaching. Overall, coral loss and shift in community structure associated with recent disturbances depended upon the relative abundance of *Acropora* to other taxa. Most notably, the site with the highest pre-bleaching coral cover and also greatest coral loss, Velidhu, was dominated by *Acropora* corals, which were largely extirpated over the course of this study. For instance, coral loss at the site Kuda Kandu was moderate compared to other sites. This site is an exposed reef, subject to strong oceanic currents, which may have moderated temperature stress. We did not however, find significant or consistent differences in levels of coral loss at sites on inner versus outer sections of atolls in the Maldives. Rather, spatial variation in the structure of coral assemblages, and their corresponding susceptibility to recent disturbances, may have been structured by differences in the disturbance regime and history among sites, whereby sites that have recovered most rapidly in the aftermath of the previous (1998) mass-bleaching are necessarily dominated by fast-growing corals^44^. Spatial variation in coral loss may also reflect differences in the occurrence of other disturbances. For example, elevated sedimentation almost certainly contributed to coral mortality at Emboodhu, especially in the shallow reef habitat, either by causing coral mortality directly^50^ or depressing coral condition and increasing susceptibility to elevated temperatures^51^.

Taxonomic variation in the bleaching susceptibility of scleractinian corals is fairly well-established^22,23,52^ and effectively accounts for the differential levels of coral loss recorded among taxa during this study. Most notably, *Acropora* corals were largely extirpated at many sites through the course of this study, as they were during the previous (1998) mass bleaching in the Maldives^44^. Following the 1998 bleaching event, branching corals (including both *Acropora* and *Pocillopora*) were reduced to less than 2% cover^44^ while other even more thermally sensitive species (e.g. *Stylophora pistillata* and *Seriatopora hystrix*) were seemingly extirpated across the entire region^47,53^. Conversely, robust corals such as massive *Porites*, which are much more resilient to elevated temperatures^24^, exhibited moderate changes in abundance through the course of the recent bleaching, and are gradually becoming the dominant corals at many sites across the Maldives. These taxonomic shifts in coral assemblages may also be exacerbated by other localised disturbances, whereby branching corals are not only most susceptible to bleaching, but also tend to be the favoured prey of crown-of-thorns starfish^54,55^ and are disproportionately affected by sedimentation^56^.

It might be expected that corals that survived prior bleaching events in the Maldives, including the major mass bleaching in 1998 and a minor bleaching event in 2010, may actually be more tolerant of elevated temperatures^22,49,57^. Repeat exposure to elevated temperatures and bleaching may either cause selective mortality, thereby effectively filtering out highly sensitive phenotypes^54^ or induce changes among survivors (e.g., shifts in Symbiodiniaceae assemblages) that increase resistance to subsequent thermal stress^44,57^. In the Maldives, it is apparent that most *Acropora* corals have recruited and grown since the last major bleaching^44^, whereas many of the other larger and longer lived corals almost certainly experienced and survived the previous mass bleaching events. There is insufficient data on individual corals or colonies to assess whether prior exposure did indeed make these corals more or less susceptible to the recent temperature stress, but prior exposure may partly account for the lower levels of mortality that occurred for these groups.

Aside from substantial declines in the abundance of corals (density of colonies, as well as overall cover), this study revealed marked changes in the structure of coral populations. Most notably, observed changes in size structure point to disproportionate loss of larger colonies across all coral taxa through the course of this study^58^, caused by high levels of partial mortality that effectively reduce colony size or increase the number of smaller colonies, and/ or differential bleaching susceptibility and mortality of large versus small colonies. Smaller coral colonies (and especially juvenile corals) are much more resistant to elevated temperatures compared to larger colonies, which is attributable to differences in flow regime and mass transfer of detrimental photosynthetic byproducts^31,23,32,33^. There is also evidence larger massive *Porites* corals may survive severe bleaching events as reported by stress bands in their skeletons^34^, however high rates of partial mortality may cause a reduction in the effective size of colonies. The altered size frequency distributions are however, concerning, especially for the faster growing corals (*Acropora* spp), because survival, growth and reproduction are strongly size dependent. The loss of larger coral colonies, and corresponding reproductive potential, is therefore, likely to greatly constrain recovery capacity^59,60^.

The selective loss of larger, reproductive coral colonies across many sites in the Maldives may have already caused suppression of population replenishment, thereby explaining low densities of juvenile corals, especially for *Acropora*. Even if large colonies survived and experienced high rates of partial mortality, rather than whole colony mortality, reductions in the effective size of colonies would impact on reproductive potential^36^. There is also evidence, that temperature stress can suppress reproductive output even among colonies that do not necessarily exhibit bleaching or partial mortality^61^.

Densities of juvenile corals recorded in October 2017 was low, especially when compared to densities of juvenile corals recorded (70.9 ± 16.7 per m^2^) at comparable sites in 2015^62^. Declines in the densities of juvenile corals may be related to the loss of larger coral colonies, and will certainly impact recovery capacity for coral populations and communities^39^. Past studies have already shown that recovery rates in the Maldives following 1998 bleaching were slow^43,44,53^, and recent disturbances will likely lead to highly protracted recovery times. With increasing frequency and intensity of disturbances and escalating effects of global climate change and human pressure, it is critical to better understand population replenishment and overall ecosystem recovery capacity and hence continue to measure juvenile density^37,63^.

The latest episode of elevated temperatures and associated coral bleaching has had substantial impacts on coral assemblages and reef ecosystems across the Central Maldives, especially given the recent occurrence of other localised but significant disturbances to corals. This latest event represents the third global-scale event since 1998^17^, reflecting the increasing importance of global climate change in structuring coral reef systems. Moreover, significant changes in coral cover and size structure of adult corals, combined with declines in densities of juvenile corals, indicate that the recovery of coral communities in the Central Maldives could be severely protracted. Time required for coral assemblages to recover following a major acute disturbance are highly variable, and may range from years to centuries^11,64,65^. In extreme cases, corals can regain pre-disturbance levels of abundance but never regain pre-disturbance structure^66^. Generally, the rate and extent of recovery depends on the severity of coral loss and on the types of corals affected. Reefs that have completely lost corals will recover much slower than reefs in which some corals survived^11,38,65^. Coral recovery also occurs faster through the growth of remnant corals rather than through settlement and subsequent growth of recruits^38^. In the Maldives, all coral taxa were severely affected, but at least some corals survived. Remnant corals were mostly larger colonies that shrank due to partial mortality. The corals will now grow to affect recovery, but extensive tissue loss and effective fragmentation of formerly large colonies will limit translocation of energy resources and thereby constrain growth and reproduction^36^. Most importantly, however, community recovery rates and trajectories will be strongly conditional upon the incidence and recurrence of further bleaching events, which are predicted to become even more frequent^67–69^, as well as other major disturbances. To minimize the severity and incidence of climate-induced coral bleaching we need to immediately and significantly reduce greenhouse gas emissions. At the same time, we need to moderate and minimize all other disturbances and pressures on coral reefs to maximize recovery and resilience of coral assemblages. In the Maldives, this requires effective action to minimize sedimentation and eutrophication associated with island reclamation and rapid expansion of resorts, which will otherwise jeopardize the reef systems necessary to sustain tourism.

## Methods

### Coral surveys

The abundance and size-structure of five dominant coral taxa (tabular *Acropora Acropora muricata*, *Acropora humilis*, *Pocillopora* spp, and massive *Porites*) were quantified in February-March 2016^44^, prior to severe mass-bleaching that occurred in April-May, 2016^13^. Surveys were repeated in October 2017 to test for changes in abundance and size structure of these corals. Surveys were undertaken at seven-10 locations across three atolls (North Ari, North and South Male atolls) in the Central Maldives Archipelago in the Indian Ocean (Fig. 1)^44^. More specifically, the abundance and size structure of corals was surveyed at seven sites in both 2016 and 2017(Fig. 1). Three new sites were added in 2017, giving a total of 10 sites (Fig. 1). At each site, three replicate 10 × 2 m belt transects were laid parallel to the reef edge, both on the reef crest (5 m depth) and slope (10 m depth).

To measure the size of individual coral colonies, we recorded only maximum diameter (to the nearest cm), which was then used to calculate the 2-dimensional projected surface area for each coral colony, following Linares *et al*.^28^. Living area (e.g., colony surface area (cm^2^)) was then calculated by subtracting the percentage of mortality for each colony. Partial mortality (percentage of tissue loss) was visually estimated *in situ* to the nearest 5% for each colony. Where a large colony had extensive partial mortality resulting in several discrete patches of living tissue, the colony was nonetheless counted as one individual.

Estimates of total coral cover and composition (relative abundance of all genera) were derived using the line transect method along tapes used to delineate each belt transect, following Hughes *et al*.^21^. Coral cover of each specific taxa was calculated based on the total intercept length of all colonies <5 cm diameter that lay directly beneath the 10 m transect line. These data were used to test for temporal changes in the abundance of individual coral taxa (mostly genera), though the limited length of line transects used here (10 m) provides limited capacity to resolve the absolute abundance of less common taxa.

To investigate recovery potential and trajectories for coral populations and communities, the abundance of juvenile corals (<5 cm maximum diameter) was recorded at all sites in October 2017. More specifically, all juvenile corals within three replicate 10 × 2 m belt transects at each depth (5 m and 10 m) and site, were identified to genus. Size (maximum diameter) of juvenile corals was also measured. It was hypothesized that larger individuals (3–5 cm) likely settled before, and survived, the recent mass-bleaching.

### Data analyses

To investigate the impacts of recent disturbances (mainly coral bleaching, but also localized outbreaks of *A*. *planci*, and sedimentation) on size structure of focal coral taxa (tabular *Acropora Acropora muricata*, *Acropora humilis*, *Pocillopora* spp, and massive *Porites*) we compared (i) the geometric mean of colony size (specifically, estimates of the projected surface area of live tissue for each coral colony), (ii) the coefficient of variation (CV) for colony size, (iii) skewness (g_1_), and (iv) kurtosis (g_2_). Separate analyses were conducted for each coral taxon. To increase resolution among smaller size classes, normalize size- frequency distributions and calculate statistics of the frequency distributions, colony surface-area data were log_10_ transformed following Bak and Meesters^70^.

These measurements were also calculated separately for each depth to test whether deeper reefs might have been less affected by recent mass bleaching and associated coral loss. Kurtosis describes the concentration of data around the central mode of a distribution among populations indicating whether the data is peaked or flat relative to the normal distribution. Positive kurtosis indicates leptokurtic distribution (e.g., peaked and highly centralized around the mean), negative kurtosis instead indicates a platikurtic distribution with a wide peak around the mean. The CV is the standard deviation as percentage of the mean and describes the variation in the data set and allows for comparisons irrespective of the mean. Skewness describes the relative abundance of colonies that are larger or smaller than the geometric mean. If the skewness is positive, the population is skewed to the right, containing a larger number of individuals in the smaller size classes. If the skewness is negative, the population is skewed to the left, with a relatively larger proportion of colonies in the larger size classes than in the smaller size classes^70^. The geometric mean provides relative measures of colony size providing information relevant to other key demographic processes, such as reproductive output^70^. Variation in the population structure of dominant coral taxa between depths (5 m versus 10 m) was investigated using 2- sample Kolmogorov–Smirnov (KS) tests for each species separately.

Differences in surface area of living tissue (SA) among sites were tested for each coral taxon using a Two-way ANOVA with mean surface area (not log-transformed) per transect as the dependent variable and depth (5 m and 10 m) and sites as independent. Difference in surface area of living tissue (SA) (not log-transformed) was also tested between 2016 and 2017 and among species using a One-way ANOVA and a Tukey’s post hoc test was then utilized to determine specific differences among species. All the assumptions were tested and met.

Variation in the extent of coral loss recorded between habitats (depths), among sites and depending on island exposure (sites on outer vs inner reefs) were tested with a *Generalized Least Squares model* (package nlme R 1.1.-14^71^). Coral cover was the dependent variable while island position, water depth, site and year were the predictors. Alternative models were compared using maximum likelihood and Akaike information criterion (AICc) (Table 1). The removal of island exposure and depth improved model fit (large AIC difference between different models) (Table 1). Likelihood Ratio Test (LRT) was also used to compare models and determine whether the increased power of the chosen model (with year and sites as predictors) was statistically significant (Table 1). Nonmetric multi-dimensional scaling (nMDS) was also used to visualize shifts in community composition from 2016 to 2017. To investigate changes in juvenile abundance between outer and inner islands, between 5 m and 10 m and between sites, a *Generalized Linear Mixed Model* with negative binomial distribution with Laplace approximation was used (package lme4 R 1.1.-14^72^). Different models were compared using a Maximum Likelihood and the model with the lowest AIC was chosen as the best representation of the variation in the data (Table 1). The best model had juvenile abundance as dependent variable and water depth, and sites as predictors. Depth was treated as fixed factor, while site was random (Table 1). However, the divergence in AIC values between all models were small (<1) suggesting that including or excluding the two predictors depth and island exposure did not strongly improve model fit (Table 1). Residuals, goodness of fit and dispersion were tested and Poisson distribution was not used because data were over-dispersed (variance was larger than mean). Finally, patterns in juvenile composition at the study sites were visualised using a nonmetric multi-dimensional scaling (nMDS). nMDSs were used as a visual tool and not to test any spatial differences in adult and juvenile community composition, however stress for each graph was reported.

All analyses were run in RStudio version 3.3.1 (RStudio Team 2015).

## Supplementary information

Supplementary Figure caption

## Data Availability

Data used in this study are available from the public data repository at the website https://figshare.com/s/d12ab2a916b3ac067780.
