# Supplementary material for: Changes in the population and community structure of corals during recent disturbances (February 2016-October 2017) on Maldivian coral reefs
Source: Sci Rep. 2019 Jun 10;9:8402. doi: 10.1038/s41598-019-44809-9 (PMC6557830; doi:10.1038/s41598-019-44809-9)
Supplement: Supplementary file 1 — Supplementary Figure caption [file 41598_2019_44809_MOESM1_ESM.docx]

**Changes in the population and community structure of corals during recent disturbances (February 2016-October 2017) on Maldivian coral reefs**

Pisapia C^1^*, Burn D^2^, Pratchett MS^2^

1 Department of Biology, California State University, 18111 Nordhoff Street, Northridge, California 91330-8303, USA

2 ARC Centre of Excellence for Coral Reef Studies, James Cook University, Townsville QLD 4811, Australia

*corresponding author: [chiara.pisapia@csun.edu](mailto:chiara.pisapia@csun.edu)

**Supplementary Information**

**Figure S1.** **Untransformed size-frequency distributions of *Acropora muricata*, tabular** *Acropora*, *Acropora humilis*, *Pocillopora* spp and *Porites* spp at 5 and 10 metres before and after the mass bleaching event.

**Figure S2. Bar graph showing composition of juvenile coral taxa at the study sites at a) 5m and b)10m.**


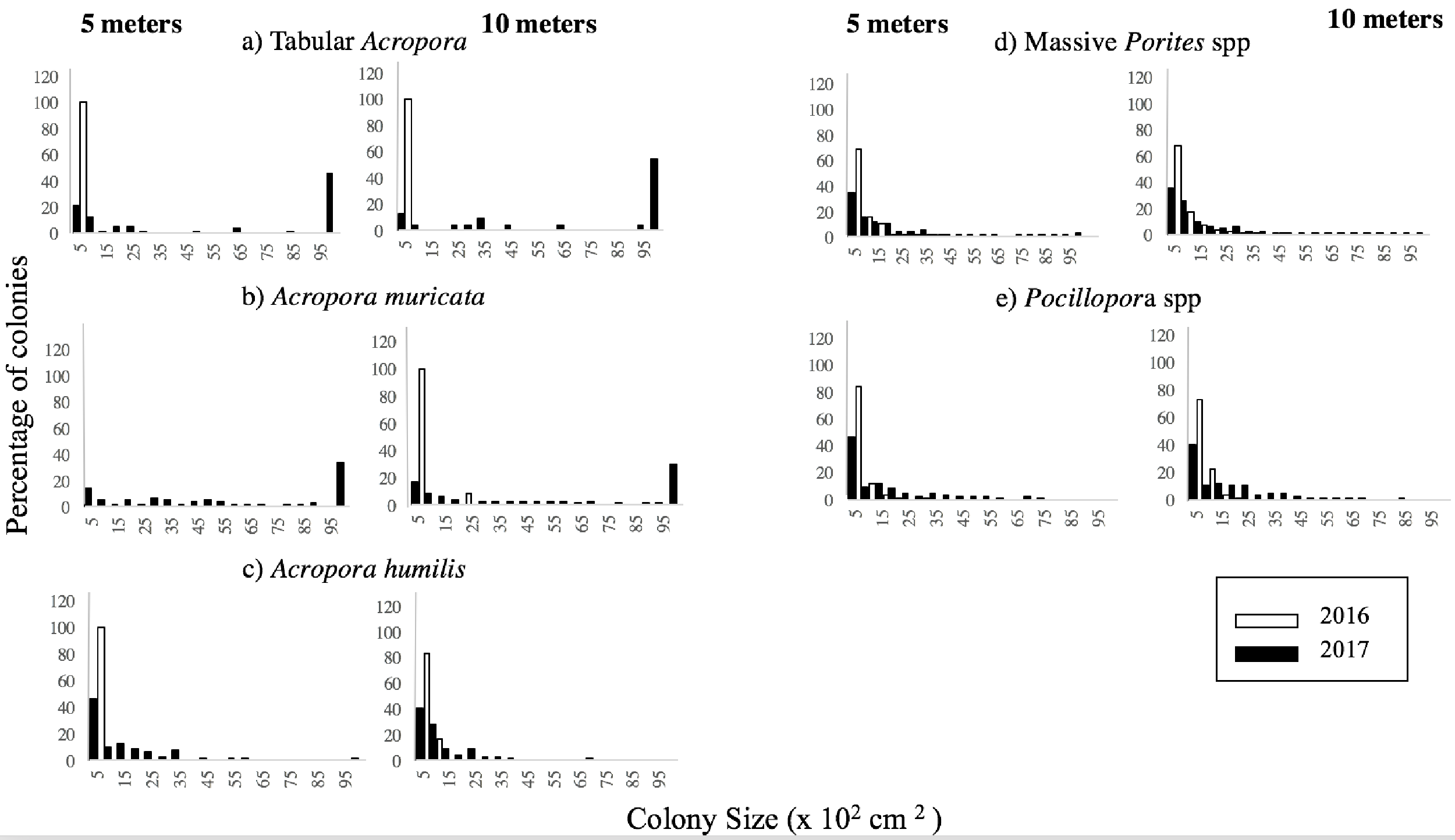





**Table S4. Summary output of the Generalized Least Squares model.** Coral cover was the dependent variable while site and year were the predictors.

|  | t-value | p-value |
| --- | --- | --- |
| Year | -7.035588 | <0.001 |
| Site Emboodhu | -0.461429 | 0.6459 |
| Site Fesdu | -0.741399 | 0.4609 |
| Site KudaKandu | -0.760808 | 0.4493 |
| Site Rasfari | 0.269068 | 0.7886 |
| Site Udafushi | -2.075055 | 0.0416 |
| Site Velidhu | 1.255196 | 0.2135 |
